# Case Report: Effect of Electroconvulsive Therapy on Obsessive-Compulsive Disorder Comorbid With Body Dysmorphic Disorder

**DOI:** 10.3389/fpsyt.2021.706506

**Published:** 2021-08-02

**Authors:** Xiaoyan Ma, Ranli Li

**Affiliations:** Institute of Mental Health, Tianjin Anding Hospital, Tianjin, China

**Keywords:** obsessive compulsive disorder, morbidity, body dysmorphic disorder, modified electroconvulsive therapy, functional impairment

## Abstract

Obsessive-compulsive disorder (OCD) is a chronic psychiatric condition that is associated with considerable morbidity, and ~90% of individuals with OCD have another psychiatric comorbidity. Patients with comorbid OCD and body dysmorphic disorder (BDD) have limited insight and poor psychosocial function, respond poorly to drug treatment, and have an increased risk of suicide. Modified electroconvulsive therapy (ECT) has been attempted to improve symptoms of OCD when drug treatment does not have a satisfactory effect. This report describes a patient who had OCD comorbid with BDD that was successfully treated with modified ECT. Although the mechanism of its effect is unclear, modified ECT may be an alternative treatment for patients with comorbid OCD and BDD. Its efficacy and mechanism of action require further investigation in a large sample of patients with these comorbid disorders.

## Introduction

Obsessive compulsive disorder (OCD) is a chronic psychiatric condition characterized by obsessive thinking, compulsive behaviors, and excessive anxiety, and is associated with considerable morbidity and economic and social burden. The obsessions and compulsions that are the hallmark of this disorder consume much time and energy and create significant functional impairment and distress (1). Approximately 3% of the general population experience OCD at some point in their lifetime (2). Moreover, epidemiological studies have found that up to 90% of individuals with OCD have a comorbid psychiatric disorder.

Body dysmorphic disorder (BDD) is a psychiatric condition characterized by a preoccupation with a perceived defect in appearance that is slight and not noticed by others. Individuals with BDD usually have poor insight and become preoccupied with the perceived physical defect. BDD is classified as an “Obsessive-Compulsive and Related Disorder” in the Diagnostic and Statistical Manual of Mental Disorders, Fifth Edition. OCD and BDD often coexist, sharing common genetic and environmental risk factors and clinical features and having a similar sociodemographic profile. Patients with OCD and BDD respond poorly to drug treatment and are at increased risk of suicide.

Selective serotonin reuptake inhibitors (SSRIs) and cognitive behavioral therapy (CBT) are the recommended first-line interventions for OCD. However, even after conventional treatment ~30–40% of patients have persistent symptoms and ongoing dysfunction (3). Patients who fail to respond to first-line pharmacological treatments and/or CBT are considered to have refractory OCD. Second-line combination, augmented, and switching strategies have been devised to customize treatment but have a limited effect on symptoms. When these strategies fail, non-pharmacological treatments may be effective.

Invasive treatments, including anterior capsulotomy, cingulotomy by thermal radiofrequency/gamma knife ablation, and deep brain stimulation, are effective therapeutic alternatives for refractory OCD, with response rates ranging from 10 to 80% (4–6). Non-invasive treatments, including transcranial magnetic stimulation and electroconvulsive therapy (ECT), have been investigated for their value in the treatment of refractory OCD. All the above-mentioned treatments have some degree of efficacy in OCD but the results of the relevant studies have been inconsistent. As yet, there is still no effective treatment for OCD, particularly for patients with complicated clinical symptoms comorbid with another psychiatric disorder. ECT is a personalized approach that holds promise as a treatment for this type of psychiatric comorbidity. Although a few reports have suggested that ECT is effective in patients with severe OCD (7, 8), the evidence is insufficient and ECT is still not recommended for OCD in the treatment guidelines (9). ECT still plays a role in the treatment of certain psychiatric disorders, particularly when the symptoms are severe and life-threatening (10, 11). Here, we report a case of OCD comorbid with BDD that was successfully treated with modified ECT.

## Case Report

An 18-year-old male high school student with an established diagnosis of OCD presented in our department accompanied by his parents after cutting off a small piece of his nose with scissors. A year and a half earlier, he had decided that his nose was ugly and started picking and prodding his nose repeatedly until it was bleeding and painful. Psychiatric treatment had been sought nearly a year before the current presentation. At that time, he was started on fluvoxamine 200 mg twice daily for 12 weeks and then in combination with CBT for a further 12 weeks. However, his symptoms worsened and he was spending increasing amounts of time picking at his nose. The fluvoxamine was switched to fluoxetine, another SSRI, which was started at a dose of 20 mg and increased gradually to 50 mg over 2 weeks, and then combined with aripiprazole 10 mg/day and alprazolam 0.8 mg/day for 12 weeks. However, the patient continued to pick at his nose.

On admission, the patient was unstable, irritable, and still repeatedly picking at his injured nose, despite being aware that he should not be doing so. He was in severe pain but still repeatedly checking his nose in a mirror. He was in obvious distress with low mood, even though he knew rationally there was nothing abnormal about his nose. On examination, he met the ICD-10 diagnostic criteria for comorbid OCD and BDD. In view of the poor efficacy of the first-line and second-line treatments, we opted for a non-pharmacological treatment in this patient.

ECT has been used successfully as a treatment for mood and psychotic disorders. Although there is no mention of ECT as a treatment modality for OCD in the guidelines (9), there are a few reports of ECT being an effective treatment for severe OCD. After a full assessment of the severity of the patient's condition and the risk of self-injury, ECT was considered as a treatment for this patient. A thorough physical examination (including measurement of blood pressure, heart rate, and respiratory function and a neurological assessment), laboratory investigations (including hematology and biochemistry), and chest radiography revealed no obvious abnormalities. Written informed consent to treatment with modified ECT under general anesthesia was obtained from the patient after he had been provided with a detailed explanation about the course of treatment and the potential risks.

Modified ECT was performed using a Thymatron System IV (America Somatics LLC, Lake Bluff, IL, USA). All doses of psychotropic drugs were reduced, fluoxetine and aripiprazole was reduced to 40mg and 5 mg/d, respectively, on the day before modified ECT. Alprazolam was discontinued the day before modified ECT. The patient was fasted for at least 8 h before ECT, and his temperature, weight, and blood pressure were measured 30min before treatment. The modified ECT procedure is performed as follows. After venipuncture with a 20–30-cm plastic needle, 10ml of 25% glucose solution are injected to confirm successful puncture. The following agents are then injected sequentially: atropine sulfate 0.5–1.0mg, diluted to 2 ml with water for injection; etomidate 0.2–0.3mg/kg, administered intravenously until loss of the eyelash reflex; 100% pure oxygen with pressure ventilation at a frequency of 20–30 tt/m, until the recovery of spontaneous breathing; and succinylcholine chloride (2mM; 100mg in glycerin) diluted to 5ml with water for injection. Approximately 30–60 s after intravenous injection of succinylcholine chloride 0.8–1mg/kg, when fasciculations in the muscle fibers of the face and limbs have stopped, a muscle relaxant is administered. Mask ventilation is used throughout the procedure with careful monitoring for airway patency and reflux aspiration and insertion of an oral protector. Frontotemporal electrodes are then placed on both sides. The stimulation parameters are as follows: charge, 101.1 millicoulombs; pulse width, 0.5 ms; frequency, 20Hz; current intensity, 0.9A; and stimulus duration, 5.6 s. A voltage of 110 V is then applied for 2–3 s. Mask ventilation was used until spontaneous respiration resumes. Finally, airway patency and thoracic movements are assessed and aspiration and auscultation are performed. Spontaneous respiration usually resumes within 5–10min, after which the intravenous needle is removed.

Our patient underwent 12 sessions of modified ECT over 3 weeks with a maximum of three sessions per week and a minimum interval between sessions of 48 h. Seizure monitoring included two-lead electroencephalography (EEG) and oximetry. The two-lead EEG electrodes were placed on the left and right frontopolar points and over the ipsilateral mastoid processes (FP1-A1 and FP2-A2 according to the International 10Y20 system). The patient did not report any adverse reactions, such as dizziness or headache, after the sessions.

Psychiatric symptoms were assessed using the Hamilton Anxiety Scale (HAM-A), Hamilton Depression Scale (HAM-D), and Yale Brown Obsessive Compulsive Scale (Y-BOCS) before and 2 and 4 weeks after completion of the course of modified ECT. Before treatment, the HAM-A, HAM-D, and Y-BOCS scores were 25, 14, and 26, respectively. After 2 weeks of treatment, there was a marked decrease in the frequency of nose-picking. The HAM-A, HAM-D, and Y-BOCS scores decreased to 17, 9, and 19, respectively, after 2 weeks of treatment and to 14, 6, and 10 at 4 weeks. At the end of the treatment course, the patient's clinical symptoms were significantly improved. Although he remained anxious about having what he perceived to be an unattractive nose, he stopped picking at it.

## Discussion

Patients with comorbid OCD-BDD have high morbidity, limited insight, and poor psychosocial outcomes (12, 13). Moreover, rates of anxiety, schizotypal features, and suicidal ideation have been reported to be higher in patients with comorbid OCD-BDD than in those with BDD or OCD alone (13, 14).

Although ECT is not recommended in the treatment guidelines for OCD, case reports indicate that it may be an effective therapy for OCD. ECT has been confirmed to be an effective treatment for depression (15) and for OCD (16). Our patient had a diagnosis of comorbid OCD and BDD. His condition continued to deteriorate on first-line therapy to the point that he sustained a self-inflicted injury. Antipsychotic medication was added without noticeable improvement in symptoms. However, after ~2 weeks of treatment with modified ECT, there was a marked improvement in his mood, and after 12 treatment sessions, the patient stopped picking at his nose and felt less anxious than before treatment.

ECT has been reported to be effective in the treatment of comorbid OCD and depression and has shown promise in the treatment of both refractory OCD (17) and severe OCD (18). Conventional OCD treatments and augmentation strategies were unsuccessful in our patient, but when combined with ECT, his symptoms improved significantly, suggesting that ECT may be effective in the treatment of comorbid OCD and BDD. According to previous reports, the beneficial effect of ECT is transient (19, 20). Our patient was discharged in remission but without any follow-up. Although it is unknown whether the favorable effect of modified ECT was transient or not in this patient, our experience in this case demonstrates that ECT may be a treatment option for OCD with comorbid BDD. However, a large-scale clinical study is needed to confirm the efficacy of ECT in patients with comorbid OCD and BDD.

The mechanism underlying the effect of ECT in patients with OCD remains unclear. Neuroimaging studies over the past two decades have found a close association between OCD and dysfunction in certain brain regions, including the orbitofrontal cortex, anterior cingulate cortex, dorsolateral prefrontal cortex, basal ganglia, caudate nucleus, and amygdala. However, circuit dysfunction is an underlying neural mechanism rather than a specific regional abnormality, as confirmed by biological and neuroimaging evidence. Dysfunction in the cortico-striatal-thalamo-cortical circuitry is strongly associated with OCD pathology. However, recent research shows that the pathological mechanism of OCD is complex and that the comorbidity rate is high regardless of subtype of OCD. Indeed, large epidemiological studies have shown that comorbidity rates in patients with OCD are generally higher than those in the general population. These comorbid psychiatric disorders add more challenges in terms of treatment. Depression is the most common comorbidity in patients with OCD, with a lifetime prevalence of 40–70% (12). Both depression and OCD are characterized by a chronic and/or recurrent course, leading to a profound deterioration in psychosocial functioning. Patients with OCD that is comorbid with another psychiatric condition have functional impairment and poor quality of life that responds particularly poorly to psychological and pharmacological treatments.

BDD is a psychiatric disorder characterized by a distressing preoccupation with a perceived defect in appearance and often coexists with OCD. A genetic study found common traits in OCD and BDD due to a 64% overlap in the genes associated with these two disorders (21). The risk of comorbid OCD and BDD is three times higher than that of OCD and BDD alone. Comorbidity may play a key role in refractory OCD in that patients with similar comorbidities might share similar important characteristics related to etiology/pathophysiology and the course of illness. Grouping patients based on their comorbidity may be a useful strategy for reducing clinical heterogeneity.

The main limitation of this report is that it is based on an isolated case and therefore cannot confirm the efficacy of modified ECT for OCD comorbid with BDD. Accumulation of more cases is needed to clarify both the pathological and etiological mechanisms of OCD. Large-scale studies are needed in the future to confirm whether or not ECT has a therapeutic effect in OCD comorbid with BDD, and if so, the mechanism involved. Another limitation may be that the patient was discharged without follow-up and the implications in terms of the durability of the beneficial effect seen.

## Conclusion

Comorbid BDD played an important role in this patient's lack of response to SSRIs and CBT as first-line therapies for OCD. Combination, augmentation, and switching strategies also achieved little improvement. Modified ECT was added to treat both the OCD and the BDD. Findings from biological research point to “circuit dysfunction” as the underlying neural mechanism for OCD rather than a specific regional abnormality. Aberrant function in the cortico-striatal-thalamo-cortical circuitry is strongly associated with the pathology of OCD. The therapeutic effect in our case suggested that modified ECT may be a good treatment option in patients with OCD and comorbid BDD. However, a clinical study in a larger sample is needed to confirm its efficacy in patients with this type of comorbidity and to elucidate the mechanism underlying its effect.

## Data Availability Statement

The original contributions presented in the study are included in the article/supplementary material, further inquiries can be directed to the corresponding author/s.

## Ethics Statement

The studies involving human participants were reviewed and approved by Medical ethics committee of Tianjin Anding Hospital. The patients/participants provided their written informed consent to participate in this study.

## Author Contributions

XM wrote the first draft of the manuscript. All authors contributed to the conception and design of this manuscript, manuscript revision, and approved the submitted version.

## Conflict of Interest

The authors declare that the research was conducted in the absence of any commercial or financial relationships that could be construed as a potential conflict of interest.

## Publisher's Note

All claims expressed in this article are solely those of the authors and do not necessarily represent those of their affiliated organizations, or those of the publisher, the editors and the reviewers. Any product that may be evaluated in this article, or claim that may be made by its manufacturer, is not guaranteed or endorsed by the publisher.

## Abbreviations

BDD: body dysmorphic disorder
CBT: cognitive behavioral therapy
ECT: electroconvulsive therapy
EEG: electroencephalography
HAM-A: hamilton anxiety scale
HAM-D: hamilton depression scale
SSRIs: selective serotonin reuptake inhibitors
Y-BOCS: yale brown obsessive compulsive scale.
